# Aquaporin-4 Dysfunction in Depression: From Pathogenic Mechanisms to Novel Therapeutic Targeting

**DOI:** 10.3390/ijms27031233

**Published:** 2026-01-26

**Authors:** Xin Xie, Hanbai Li, Yanfen Chang, Meijiao Ji, Mengqi Wang, Jiahao Hu, Hui Sheng

**Affiliations:** College of Basic Medical Sciences, Naval Medical University, Shanghai 200433, China; xx2022043@163.com (X.X.);

**Keywords:** AQP4, depression, glutamate, neuroinflammation, mitochondria

## Abstract

Depression represents a leading cause of global disability, yet its pathogenesis remains incompletely understood. This review synthesizes emerging evidence highlighting the multifaceted role of Aquaporin-4 (AQP4), the central nervous system’s predominant water channel, in the pathophysiology of depression. Preclinical studies frequently report AQP4 dysregulation in depression models, characterized by reduced perivascular expression and impaired polarization in mood-relevant brain circuits. We delineate how AQP4 impairment is implicated in depression through several interconnected mechanistic pathways: (1) exacerbating glutamate excitotoxicity by disrupting astrocytic glutamate clearance; (2) impairing monoaminergic neurotransmission and synaptic plasticity; (3) potentiating neuroinflammatory cascades; (4) inducing mitochondrial functional impairment and oxidative stress; and (5) participating in hypothalamic–pituitary–adrenal (HPA) axis dysregulation by disrupting perineuronal osmotic and ionic homeostasis in response to arginine vasopressin (AVP) signaling. Furthermore, we explore the therapeutic relevance of AQP4, noting that diverse antidepressant treatments appear to partly exert their effects by modulating AQP4 expression and function. Collectively, the evidence positions AQP4 not as a solitary causative factor, but as a critical contributing component within the broader astrocyte–neuron–immune network. We therefore propose AQP4 as a promising node for therapeutic intervention, whose modulation may help counteract core pathophysiological processes in depression, offering a potential avenue for novel treatment development.

## 1. Introduction

Depressive disorder represents a pervasive psychiatric condition affecting over 300 million people worldwide, characterized by pervasive anhedonia, cognitive impairment, and somatic comorbidities that frequently confer treatment resistance [1,2]. Critically, depression constitutes the foremost modifiable risk factor for suicide, accounting for approximately 28% of global suicide-attributable disability-adjusted life years according to World Health Organization meta-analyses [3,4]. The disease burden is projected to escalate dramatically, with major depressive disorder (MDD) anticipated to become the leading cause of global disease burden by 2030—surpassing. cardiovascular conditions and malignancies [3]. This trajectory underscores an urgent need to move beyond traditional concepts of depression and elucidate novel pathophysiological mechanisms. While monoaminergic neurotransmission deficits have dominated therapeutic development for decades, their explanatory limitations are increasingly apparent [5,6]. A fundamental challenge remains the incomplete understanding of the disease’s pathogenesis.

Aquaporin-4 (AQP4), the predominant water channel in the central nervous system (CNS), exhibits polarized localization at perivascular astrocyte end-feet where it orchestrates glymphatic clearance, ion homeostasis, and astrocytic physiology [7,8,9]. Beyond these canonical roles, accumulating evidence demonstrates AQP4’s critical involvement in regulating neuroinflammatory responses [10], glutamate recycling [11], synaptic plasticity [12], etc. Notably, AQP4 dysregulation contributes to the pathogenesis of diverse neurological disorders including Alzheimer’s disease (AD) [13], stroke [14], and cerebral edema [15]. More recently, growing recognition implicates AQP4 dysregulation in the pathophysiology of depression [16]. This review systematically synthesizes current evidence linking AQP4 impairment to depression pathogenesis through multiple mechanistic pathways and evaluates its therapeutic relevance. We aim to consolidate existing research findings and provide an integrated framework for understanding AQP4’s multifaceted contributions to depressive disorders, thereby identifying promising therapeutic targets.

## 2. Molecular Structure and Localization of AQP4

In the brain, the only aquaporins with well-established physiological and pathological roles are AQP1, AQP4, and AQP9 [17]. First identified by Peter Agre in 1994, AQP4 is the most extensively characterized astrocyte-associated water channel [18]. Structurally, the AQP4 monomer (approximately 30 kDa) consists of six transmembrane α-helices (S1, S2, S4, S5, S6, S8) and two shorter, partially membrane-embedded helices (S3, S7), with each monomer forming an independent water-selective channel [19,20]. High-resolution X-ray crystallographic analyses reveal the presence of a narrow selectivity filter (diameter ~1.5 Å) within the channel [21]. This filter incorporates the conserved NPA (Asn-Pro-Ala) motifs and key residues such as Arg216 and His201 [21]. Through a combination of steric hindrance and electrostatic repulsion mechanisms, this structure ensures high selectivity for water molecules while effectively preventing the permeation of protons, ions, and other solutes [21]. Functionally active AQP4 assembles in the plasma membrane as homotetramers or heterotetramers [22]. Notably, a central pore is formed at the core of each tetramer, which has been proposed to potentially facilitate the transport of gases (such as CO_2_ and NH_3_) or ions [23]. However, the physiological relevance of transport through this central pore remains a subject of ongoing debate. These tetramers can further self-assemble into lattice-like supramolecular structures known as orthogonal arrays of particles (OAPs), a process primarily regulated by two major transcript isoforms, M1 and M23 [24,25]. The full-length M1 isoform possesses an extended N-terminus that inhibits large OAP assembly, while the shorter M23 isoform drives OAP formation through hydrophobic interactions [25,26]. Their co-expression and formation of heterotetramers in vivo determine the ultimate size and stability of OAPs [27].

AQP4 exhibits a highly polarized distribution pattern in astrocytes, with particular enrichment at critical brain-fluid interfaces: including the perivascular endfoot membranes of the blood–brain barrier, the pial/subpial endfeet at the cerebrospinal fluid (CSF)-parenchyma interface, and the ependymal/subependymal processes surrounding the ventricular system [28,29]. This specific localization is mediated by an anchoring system, primarily via the dystrophin-associated protein complex and its component α-syntrophin, which bridges AQP4 to the extracellular matrix at the basal lamina [30,31,32,33]. Genetic deletion of key components like dystrophin or α-syntrophin leads to a dramatic loss of perivascular AQP4 polarization [32,34]. Regional specificity analysis reveals that AQP4 density is highest in perivascular, subarachnoid, and periventricular astrocytes, and is also markedly elevated in osmosensitive regions (such as the supraoptic nucleus and subfornical organ), consistent with its core function in maintaining water and ion homeostasis [29,35,36]. Recent studies further suggest that sparse but functional pools of AQP4 are also present on the perisynaptic membranes of astrocytes at tripartite synapses, facing neurons [37]. This indicates that AQP4 may extend beyond its traditional role in fluid balance, possessing potential for direct synaptic modulation and the regulation of neural plasticity.

## 3. Foundational Physiological Roles of AQP4 in the CNS

AQP4 is the most abundant and distinctive water channel in the CNS [38]. Its functions extend well beyond passive water transport, positioning it as a core executor of astrocyte activity and a critical regulator of brain homeostasis [39,40]. By modulating the dynamic equilibrium of water, ions, and cellular volume, AQP4 establishes a molecular foundation for the stability of the neuronal microenvironment, thereby influencing neural signaling and plasticity [31]. A comprehensive understanding of these fundamental roles provides the necessary framework for deciphering its involvement in pathological conditions such as cerebral edema, epilepsy, and neurodegenerative diseases. 

### 3.1. Molecular and Cellular Basis: Facilitated Water Transport and Osmotic Balance

The primary function of AQP4 is to serve as a bidirectional, water-selective pore [38]. It assembles as a tetramer, with each monomer forming an independent water channel, a structure essential for maintaining baseline brain water homeostasis [41]. Under physiological conditions, AQP4 mediates continuous, subtle water exchange between CSF, the interstitial space, and across vascular interfaces [42]. For example, in response to osmotic changes, astrocytes rapidly adjust their volume via AQP4, buffering acute shifts in extracellular osmolality and protecting neurons from osmotic stress [43]. Foundational studies show that AQP4-knockout (AQP4^−^/^−^) mice exhibit a slight but significant increase in baseline brain and spinal cord water content compared to wild-type littermates, directly underscoring its role in the fine-tuning of physiological water balance [44].

### 3.2. Potassium Spatial Buffering and Ionic Homeostasis

A key physiological function of AQP4 is its cooperation with the astrocytic inward rectifier potassium channel Kir4.1 to regulate extracellular potassium (K^+^) spatial buffering [45]. Neuronal activity releases K^+^ into the restricted extracellular space, elevating local concentration ([K^+^]_0_) and potentially disrupting neuronal excitability [46]. Astrocytes uptake excess K^+^ mainly via Kir4.1 [47]. The accompanying K^+^ influx creates a local osmotic gradient that drives water entry into astrocytes through AQP4. This coupled transport is crucial for maintaining extracellular volume and ionic stability. In AQP4^−^/^−^ mice, the clearance rate of extracellular K^+^ following stimulation is significantly slowed, impairing K^+^ buffering and affecting neuronal excitability, which illustrates how AQP4 influences basic neuroelectrical activity [48].

### 3.3. Modulation of the Perisynaptic Microenvironment

Within the tripartite synapse—comprising pre- and postsynaptic neurons along with perisynaptic astrocyte processes—AQP4 contributes to microenvironmental regulation [37,49]. Neurotransmitter release and reuptake involve rapid local osmotic and ionic shifts [50]. Perisynaptic AQP4 is believed to regulate water flux in this microdomain, influencing extracellular geometry and diffusion properties, thereby indirectly shaping the spatiotemporal dynamics of neurotransmitters. AQP4 deficiency is associated with reduced astrocytic glutamate uptake, possibly due to altered transporter function or microenvironmental changes [51]. Moreover, AQP4-dependent volume adjustments can modulate synaptic cleft concentrations of neurotransmitters and neuromodulators, supporting synaptic plasticity mechanisms such as long-term potentiation (LTP) and depression [52]. Behaviorally, AQP4^−^/^−^ mice show impairments in spatial learning and memory consolidation, linking AQP4 to higher cognitive functions [12,53].

### 3.4. Polarized Localization at Fluid Interfaces: Blood-Brain and CSF Barriers

The highly polarized enrichment of AQP4 on astrocyte endfeet membranes underpins its systemic functions [54]. Via interaction with anchoring proteins such as α-syntrophin within the dystrophin-associated complex, AQP4 is specifically targeted to endfoot domains facing capillary basal laminae and pial/ependymal surfaces [30]. This strategic placement positions AQP4 at critical brain-blood and brain-CSF interfaces [55,56], where it not only mediates trans-barrier water movement but may also participate in neurovascular coupling. Evidence suggests that osmotic stimuli or activity-induced astrocytic Ca^2+^ signals may, through AQP4 or associated channels like TRPV4, modulate vascular tone and local cerebral blood flow [43,57].

### 3.5. The Glymphatic System and Metabolic Clearance

The glymphatic system hypothesis expands the role of AQP4 to a whole-brain clearance mechanism [58]. It proposes that, particularly during states such as sleep, CSF enters the brain along para-arterial spaces [59]. Water from this influx moves into the extracellular space via AQP4 on astrocyte endfeet, driving convective interstitial fluid flow that promotes the clearance of metabolic wastes along para-venous pathways [59]. Although hydrodynamic details remain debated, AQP4 is central to this proposed clearance model [60]. AQP4^−^/^−^ mice exhibit significantly reduced interstitial solute clearance and increased amyloid-β deposition [59,61], providing physiological evidence for AQP4’s role in maintaining brain metabolic homeostasis.

## 4. AQP4 and Depression

Converging evidence from clinical and preclinical animal studies substantiates AQP4 dysregulation as being implicated in the pathophysiology of MDD. The following table summarizes recent studies on the association between AQP4 and depression, most of which demonstrate a significant association between AQP4 and depression or depression-related diseases, but there are also studies with opposing opinions, proving that the relationship between AQP4 and depression remains to be investigated (Table 1 and Table 2). Histopathological analyses reveal significantly reduced perivascular AQP4 immunoreactivity (indicating impaired astrocyte end-foot coverage) in corticolimbic vasculature of MDD patients [62,63]. Complementary transcriptomic investigations demonstrate downregulation of AQP4-associated mRNA transcripts in depressive cohorts, suggesting compromised channel expression at the transcriptional level [64]. Postmortem validations further identify region-specific AQP4 deficiency, with marked reductions in both mRNA and protein expression particularly affecting mood-regulating gray matter structures—including the hippocampus (dentate gyrus and cornu ammonis), locus coeruleus, and prefrontal cortex-while sparing white matter tracts [16]. This anatomically selective AQP4 deficiency underlies a disruption in brain fluid homeostasis, including glymphatic function, which in turn may contribute to disease progression by causing impaired metabolic waste clearance, aberrant distribution of neurotransmitters, and altered neuroimmune communication.

Complementary preclinical evidence also establishes AQP4 dysregulation as causally contributing to depression pathogenesis. Chronic unpredictable mild stress (CUMS) models demonstrate impaired AQP4 polarization and reduced transcript levels of AQP4 and dystrophin-associated complex components (agrin, laminin, dystroglycan) in anterior cortex, concomitant with glymphatic dysfunction [65]. These pathological alterations are reversibly rescued by antidepressant therapies or glucocorticoid receptor antagonism [65]. Parallel CUMS exposure significantly decreases AQP4 protein expression in hippocampal dentate gyrus and choroid plexus—effects prevented by mood stabilizers [66]. Additional stress paradigms (anxiety-provoking stimuli, gestational stress) consistently replicate cortical AQP4 downregulation [66,67]. Crucially, genetic ablation studies confirm causal involvement: AQP4 knockout (AQP4-KO) mice exhibit exacerbated hippocampal neurogenesis impairment and astrocytic vulnerability during chronic corticosterone exposure, with significant aggravation of depression-like behaviors [68]. Lipopolysaccharide (LPS)-induce animal model of depression further demonstrated that perivascular AQP4 redistribution, establishing consistent AQP4 dysregulation across etiologically diverse depression models [69].

**Table 1:** Association of AQP4 with depression-related disorders in animal studies.

Material Type	Experimental Paradigms	Experimental Models	Key Outcomes	References
Male and female C57BL/6 J mice (6–8 weeks of age). Adeno-associated virus 5 (AAV5) or a scrambled control (AAV5-scrambled-shRNA). Prefrontal cortex tissue	Stress-based models of depression	Chronic Unpredictable Stress (CUS) Model	CUS induced pronounced astrocyte dystrophy, loss of vascular AQP4 coverage, and working memory deficits in male mice	[70]
Adult male C57BL/6 mice (10–12 weeks old) Melatonin, TGN020, artificial CSF tracers and antibodies for immunohistochemistry Primary astrocyte cultures ELISA kits, qPCR reagents	Stress-based models of depression	CUMS mouse model	CUMS disrupted AQP4 polarization (reduced M23/M1 ratio) at astrocytic endfeet	[71]
Adult male C57BL/6 mice (9–11 weeks) and CD1 mice (9–13 months) Primary hippocampal neurons isolated from embryonic day 18–19 C57BL/6 mice NMDA (100 μmol/L), KN-93 (CaMKII inhibitor, 1 μM); TBB (CK2 inhibitor, 50 μM); Rapamycin (autophagy activator, 100 nM); Fluoxetine (10 mg/kg).	Stress-based models of depression	chronic social defeat stress Mouse Model	AQP4 Upregulation in Depression AQP4 Knockdown Ameliorates Depression	[72]
Adult male C57BL/6 mice (10–12 weeks old) Polyunsaturated fatty acid (PUFA) supplement, escitalopram (Es, 10 mg/kg, intraperitoneal); tracers (FITC-dextran 3 kDa, Texas Red-dextran 40 kDa)	Stress-based models of depression	CUMS mouse model	CUMS caused AQP4 depolarization in astrocytic endfeet; PUFA restored perivascular AQP4 localization, supporting glymphatic flow. Es partially increased AQP4 expression but did not correct polarization.	[73]
Thirty-eight specific pathogen-free C57BL/6 mice (male, age unspecified) Chemical Reagents Gadolinium-diethylenetriamine pentaacetate (Gd-DTPA) as paramagnetic contrast agent; Texas Red-dextran-3 (3 kDa); immunofluorescence antibodies	Stress-based models of depression	CUMS mouse model	Immunofluorescence confirmed the down-regulation of AQP4 expression in CUMS mice	[74]
Male Wistar rats (6 weeks old, ~200 g; *n* = 80) Lithium chloride (2.5 mEq/kg, i.p.); Gd-DTPA (0.5 mmol/kg) anti-AQP4, anti-GFAP, anti-claudin-5, anti-BDNF, anti-IL-6	Stress-based models of depression	Chronic mild stress (CMS) rats model	Lithium attenuated CMS-induced reduction in hippocampal AQP4/GFAP ratio	[75]
Adult male AQP4-KO (AQP4−/−) and wild-type (AQP4+/+) mice (2 months old, CD1 background). Fluoxetine hydrochloride (10 mg/kg, i.p.), bromodeoxyuridine Antibodies for immunohistochemistry	Stress-based models of depression	CUMS mouse model	Ketamine increased the AQP4 expression in the hippocampus of the CUMS mice	[76]
Wistar rats (male and female) at postnatal days 15 (juvenile) and 70 (adult) Sodium fluorescein (NaF, 376 Da) RT-qPCR ELISA kits	Stress-based models	Early-life stress (ELS) Model: Maternal separation (MS) protocol	MS upregulated AQP4 expression exclusively in the dSTR of juvenile males	[77]
Male C57BL/6 mice (8 weeks old) Antibodies for immunofluorescence ELISA kits	Depression-like behavior based on neuroinflammation	High-fat diet (HFD)-induced obesity in mice	HFD-induced obese mice have reduced AQP4 expression in the caudo-putamen nucleus and anterior cingulate cortex	[78]
Male and female C57BL/6 mice (8–10 weeks old; n = 6–12/group) Antibodies for immunofluorescence ELISA kits qPCR reagents	Depression-like behavior based on neuroinflammation	Traumatic brain injury (TBI) Model: Lateral fluid percussion injury in mice to induce moderate TBI	TBI reduced AQP4 polarization at 3 DPI, indicating glymphatic impairment.	[79]

**Table 2:** Association of AQP4 with depression-related disorders in human studies.

Material Type	Disease Area	Experimental Models	Key Outcomes	References
DNA isolated from peripheral blood samples of 342 patients with confirmed coronary artery disease (CAD). Genome-wide genotyping data Demographic and psychiatric assessment data	Vascular depression, a subtype of late-onset depression Patients with atherosclerotic CAD	Genome-wide association study	The genome-wide significant variant rs528732638 is associated with a 3.6-fold increase in the odds of lifetime depression The genomic region harboring rs528732638 acts as an expression quantitative trait loci (eQTL), regulating the expression of the AQP4 gene	[80]
50 patients with a major depressive episode (MDE) (25 with MDD, 25 with bipolar disorder [BD]) and 30 matched healthy controls (HCs)	MDE in unipolar MDD and BD Anxiety disorders, Personality disorders Treatment-resistant depression	Human Clinical Cohort	No serum AQP4 autoantibodies detected in any MDE patients (MDD or BD) or HCs at baseline or follow-up	[81]
57 patients with optic neuritis Demographic information (age, sex), clinical metrics (visual acuity in better- and worse-seeing eyes, number of recurrence events), and patient-reported outcome measures Serum AQP4-IgG antibody status	AQP4 antibody-seropositive optic neuritis (AQP4-ON) Visual impairment Depression risk	Human clinical cohort model	AQP4-ON was significantly associated with a lower the 25-item National Eye Institute Visual Function Questionnaire (VFQ-25) composite score compared to idiopathic ON (ION) AQP4-ON was significantly associated with a higher the Beck Depression Inventory-II (BDI-II) score compared to ION	[82]
31 patients with stress-induced exhaustion disorder (SED), 31 with MDD, and 61 HCs Anti-AQP4 DyLight 488, anti-glial fibrillary acidic protein (GFAP) DyLight 755, anti-CD41-FITC (platelet marker), and anti-CD154-PE (CD40 Ligand); Isotype-matched immunoglobulins	SED MDD	A cross-sectional study	SED patients showed significantly higher concentrations of AQP4+ EVs MDD patients had higher AQP4/GFAP+ EVs than HCs, but lower than SED	[83]
Brain samples from subjects with MDD and matched non-psychiatric controls Antibodies for immunohistochemistry molecular biology tools for qPCR ELISA kits Rodents (rats and mice)	MDD	Human Postmortem Studies	The coverage of blood vessels by AQP4-positive astrocytes in MDD is reduced. Downregulation of AQP4 in MDD	[84]

As above mentioned, investigations into AQP4 expression in depression models report seemingly paradoxical findings, including regional downregulation, upregulation, or altered polarization. These discrepancies are not necessarily inconsistent; rather, they underscore the context-dependent nature of AQP4 dysregulation, influenced by factors such as brain region, disease subtype, stress paradigm, and the specific molecular parameter altered.

The brain region under investigation is a primary determinant of AQP4 expression changes. Consistent downregulation of AQP4 has been observed in emotion-processing gray matter structures, such as the hippocampus and prefrontal cortex, both in post-mortem studies of MDD patients and in chronic stress models like CUMS [63,65]. This regional vulnerability may be linked to high metabolic demand and susceptibility to excitotoxic and inflammatory insult. Conversely, distinct pathological contexts, such as the neuroinflammatory response following TBI, may be associated with disrupted polarization of AQP4 at astrocytic endfeet without an overall change in total protein levels [79]. This suggests that the loss of polarized localization—critical for glymphatic function—can occur independently of changes in total AQP4 expression, a nuance that may explain conflicting reports from studies employing different methodologies.

The nature of the depressive insult also critically influences AQP4 dynamics. CMS or CUMS protocols typically lead to reduced hippocampal AQP4 expression and polarization, supported by DCE-MRI evidence of glymphatic dysfunction [74]. However, acute or severe stressors may trigger different adaptive or maladaptive responses [85]. Furthermore, comorbidity plays a significant role. Diet-induced obesity, a common comorbidity of depression, can induce frontal-striatal gliosis and is associated with reduced AQP4 levels, suggesting that metabolic stress engages pathways converging on AQP4 downregulation [78].

The total abundance of AQP4 protein and its polarized localization to perivascular astrocytic endfeet can be differentially regulated with distinct functional consequences. A reduction in total AQP4 expression, as observed in the hippocampus following chronic stress, directly impairs the channel’s water transport capacity [65]. Conversely, mislocalization or depolarization of AQP4 can disrupt glymphatic flow without necessarily altering total protein levels, as illustrated by studies on sleep disruption and TBI [79]. Depolarization of AQP4 distribution has also been observed in many models of depression [73,86]. Therefore, studies reporting no change in total AQP4 levels cannot rule out significant functional impairment at the glymphatic interface due to loss of polarization.

Consequently, seemingly contradictory findings across studies reflect the complex, multifaceted regulation of AQP4. This is not a simple model of loss- or gain-of-function, but rather positions AQP4 dysfunction as a final common pathway reachable via various etiologies, each leaving a distinct molecular signature on AQP4 expression and localization. Future research must explicitly account for these variables by systematically analyzing both polarization status and total expression across different brain regions and disease models to comprehensively elucidate the role of AQP4 in the pathophysiology of depression. This refined understanding positions AQP4 not merely as a biomarker of dysfunction but as a central, dynamically regulated node interfacing neurovascular, glymphatic, and inflammatory processes—a promising target for mechanistically informed therapeutic strategies.

## 5. Potential Mechanisms Underlying the Contribution of AQP4 to Depression

### 5.1. Glutamate Excitotoxicity

Glutamate excitotoxicity has emerged as a critical pathogenic mechanism underlying depression [87]. It has been demonstrated that sustained elevation of extracellular glutamate concentrations and aberrant N-methyl-D-aspartate receptor (NMDAR) activation occur in corticolimbic circuits of depressed patients and chronic stress models [88,89]. Critically, impaired astrocytic glutamate clearance initiates excitotoxic cascades, which may underlie core depressive symptomatology [90].

Emerging evidence indicates that AQP4 expression can modulate cerebral glutamate homeostasis, with deficiency being linked to excitotoxic risk in various neurological conditions [91]. In AQP4-KO mice, across different models have demonstrated significantly elevated extracellular glutamate concentrations and NMDAR-mediated excitatory postsynaptic currents are significantly elevated in the nucleus accumbens and hippocampus [92]. Research suggests that these perturbations correlate with behavioral despair phenotypes relevant to depression.

Mechanistically, experimental evidence from cell and animal models indicates that AQP4 deficiency can impairs astrocytic glutamate clearance, evidenced by reduced [^3^H]D,L-glutamate uptake in cortical astrocytes and decreased glutamate transporter activity in primary cultures, which paradoxically attenuates glutamate-induced cytotoxicity due to compensatory upregulation of antioxidant pathways (e.g., Nrf2/ARE) [93]. Critically, AQP4 co-localizes with the astrocytic glutamate transporter-1 (known as EAAT2 in humans and GLT-1 in rodents) at perivascular endfeet domains, suggesting the formation of a functional complex that could synchronize water efflux with glutamate translocation via osmotic gradients [11,94,95,96]. Consistent with this spatial organization, AQP4 loss has been shown to downregulate GLT-1 expression in a region-specific manner and suppresse glutamate uptake in primary astrocytes by disrupting transporter membrane trafficking [91,97]. This regional heterogeneity may contribute to the differential vulnerability of brain circuits to excitotoxic injury observed in various disorders, including depression. In addition, heterologous AQP4 expression in HEK-293 cells was found to upregulate transgenic EAAT2 protein, an effect abrogated by blockade of calcium-dependent PKC signaling, implying a potential mechanism through which AQP4 might regulate transporters via kinase-mediated phosphorylation [98]. Collectively, data from preclinical models suggest that AQP4 deficiency could promote elevated extracellular glutamate via impaired transporter function and expression, potentially inducing NMDAR hyperactivation that is associated with dendritic spine loss in prefrontal pyramidal neurons—a structural hallmark also observed in chronic stress models. This body of work establishes excitotoxicity as a potential pathophysiological mechanism that may link astrocyte dysfunction to depressive pathogenesis.

### 5.2. Impaired Neurotransmission

Impaired neurotransmission constitutes a pathophysiological cornerstone of depression, characterized by dysregulated monoaminergic and glutamatergic signaling across corticolimbic-hypothalamic circuits [99]. These deficits manifest as synaptic failure—evidenced by dendritic atrophy, spine loss, and disrupted plasticity—compromising neural connectivity essential for emotion regulation [100,101]. Thus, synaptic dysfunction serves as a convergent mechanism linking distributed network disruption to core depressive symptomatology.

AQP4 has been implicated in modulating monoaminergic neurotransmission and synaptic plasticity, suggesting a potential role in depression-related neural circuitry [102,103,104,105]. Prior studies, primarily in addiction or other neurological models, demonstrate that AQP4 deletion attenuates dopamine dynamics in reward pathways: it blunts morphine-induced dopamine elevation in the nucleus accumbens and reduces cocaine-evoked extracellular dopamine release compared to wild-type controls [105,106]. Furthermore, research in neurodegeneration models indicates that AQP4 deficiency exacerbates dopaminergic neurodegeneration in the substantia nigra following α-synuclein fibril inoculation, potentially through impaired clearance of neurotoxic glutamate and α-synuclein aggregates via glymphatic dysfunction [107]. Mechanistically, studies suggest that AQP4 may mediate estrogenic regulation of serotonergic function, as evidenced by abolished hippocampal 5-HT increases in AQP4-KO mice upon estrogen administration, proposing that AQP4-dependent water flux facilitates 5-HT1A receptor signaling through modulation of perisynaptic space geometry [108].

Critically, AQP4 is thought to influence synaptic plasticity, which is essential for cognitive-emotional processing. Observations from neuroimmune disorders, such as the impaired neuroplasticity in patients with AQP4 antibody-positive neuromyelitis optica spectrum disorder, indicate a potential link [109]. While its precise mechanisms remain incompletely characterized, AQP4-KO mice exhibit impaired LTP in key depression-relevant circuits [12,49]. Preclinical evidence also suggests that AQP4 loss can reduce lactate shuttling via monocarboxylate transporters, which may deprive neurons of energy substrates for plasticity maintenance [110]. Furthermore, chronic stress downregulates hippocampal AQP4 expression by 40–60% in rodent models [111], a change that coincides with LTP impairment and dendritic simplification—phenotypes that have been rescued by AQP4 overexpression in some models [52]. Collectively, these findings from various experimental systems position AQP4 dysfunction as a potential contributing event in depression pathophysiology. It may contribute to the disorder by affecting critical processes including monoaminergic and glutamatergic neurotransmission, as well as synaptic plasticity, which could ultimately converge on the synaptic deficits that underlie core depressive symptomatology.

### 5.3. Neuroinflammation

Major advances in depression pathophysiology research have established neuroinflammation as a fundamental pathogenic driver [112]. Convergent clinical and preclinical evidence now implicates maladaptive neuroinflammatory activation in the etiology and progression of both human depressive disorders and experimental models of depression [113,114]. Key pathological features include microglial priming [115], elevated pro-inflammatory cytokines [116], and inflammasome activation within corticolimbic circuits [117], which may induce depressive symptomatology.

To date, only a limited number of studies have investigated the relationship between AQP4, neuroinflammation, and depression. The existing evidence, primarily from animal models of depression, has converged to indicate several key disturbances: a depolarized expression of AQP4, significant glymphatic dysfunction, and a concomitant upregulation of neuroinflammatory markers [73]. Notably, increasing studies utilizing AQP4-KO mice demonstrate a critical role for AQP4 in modulating neuroinflammatory responses across diverse brain pathologies. For instance, studies in models of Parkinson’s disease have shown thatAQP4 deficiency disrupts the balance of inflammatory cytokines in the midbrain, evidenced by exacerbated reactive gliosis (increased astrocytosis and microgliosis), activation of the nuclear factor κB (NF-κB) pathway, and elevated production of interleukin-1β (IL-1β) and tumor necrosis factor-α (TNF-α), both under basal conditions and following 1-methyl-4-phenyl-1,2,3,6-tetrahydropyridine intoxication [10]. Similarly, research in ischemic stroke models, such as Shi et al. reported that AQP4-KO mice exhibit heightened cellular inflammation after middle cerebral artery occlusion, characterized by neutrophil infiltration within the ischemic core, microglial activation in the peri-infarct boundary zone, and upregulation of the proinflammatory receptors cysteinyl leukotriene receptors (CysLT1 and CysLT2) in injured neurons [118]. These findings collectively hint at a potential inhibitory function for AQP4 in post-ischemic inflammation regulatory role of AQP4 in inflammatory responses after brain injury. Furthermore, in APP/PS1 transgenic mouse models of AD, AQP4 deletion exacerbates Aβ accumulation, a key driver of neuroinflammation via microglial immune activation and induction of neurotoxic astrocyte phenotypes [119,120].

Accumulating evidence from models of sleep disturbance also indicates that AQP4 deficiency may aggravate the activation of the NF-c pathway and NLRP3 inflammasomes, as reflected by elevated levels of NLRP3 and ASC in the hippocampus following chronic sleep disruption [121]. This impairment may potentially stem from disrupted glymphatic function due to AQP4 dysregulation, which could contribute to the accumulation of inflammatory cytokines and metabolic waste, thereby possibly perpetuating a vicious cycle of neuroinflammation. Despite the current absence of direct evidence connecting AQP4 to depression through neuroinflammation, deciphering their intricate interplay is nevertheless key to unraveling the disease mechanisms and paving the way for novel therapies.

### 5.4. Mitochondrial Dysfunction

Mitochondrial dysfunction constitutes a pathogenic nexus in depression, driving bioenergetic collapse and oxidative stress within corticolimbic circuits [122]. Key manifestations, including impaired electron transport chain activity (ATP deficit), accumulated mtDNA deletions, and aberrant fission-fusion dynamics, converge to disrupt synaptic resilience and stress adaptation, which are core features of depressive pathophysiology [122,123]. Critically, mitochondrial-targeted agents reverse depressive phenotypes in preclinical models, confirming causal involvement in disease etiology [124].

AQP4 dysfunction may disrupt mitochondrial homeostasis through multi-mechanistic pathways relevant to depression pathogenesis. Research in Alzheimer’s disease (APP/PS1) models has shown that AQP4 deficiency exacerbates hypothalamic mitochondrial respiratory chain impairment, correlating with aberrant AQP4 polarization and reduced Complex I activity—a critical defect in NADH oxidation [125]. Intriguingly, studies in AQP4-KO astrocytes exhibit fragmented mitochondrial networks and impaired Ca^2+^ buffering capacity, which in co-culture systems directly compromises neuronal bioenergetics [126,127]. Evidence from models like OVX/D-galactose treatment suggests that AQP4-KO induces oxidative stress cascades, as evidenced by reductions in T-SOD/T-AOC and elevated MDA, with prefrontal cortex mitochondria showing greater ROS susceptibility than hippocampal counterparts, a pattern which aligns with regional vulnerability in depression [127]. Simultaneously, retinal bioenergetic suppression (decreased Pgc1α, CoxIV, CytC) and fission-fusion imbalance (decreased Fis1/Mfn1/Mfn2) have been found to coincide with disrupted glycolytic-astrocyte lactate shuttle, potentially depriving neurons of plasticity substrates [128]. Notably, experiments indicate that mitochondrial fission inhibitor mdivi-1 attenuates hypoxia-induced AQP4 upregulation via NF-κB-dependent crosstalk, while AQP4 overexpression rescues stress-induced mitochondrial elongation in medial prefrontal cortex astrocytes, normalizing ATP production by 80% [129]. Collectively, findings from diverse experimental systems suggest that AQP4 serves as an astrocytic gatekeeper of mitochondrial integrity, whose impairment may drive depressive pathophysiology through amplified oxidative stress and bioenergetics collapse.

### 5.5. HPA Axis Dysregulation

The pathogenesis of depression is intricately linked to dysregulation of the hypothalamic–pituitary–adrenal (HPA) axis, in which the key neural circuit formed by the prefrontal cortex (PFC) and the basolateral amygdala (BLA) plays a central role [130,131]. Chronic stress disrupts the balance of this circuitry, leading to dendritic hypertrophy and functional hyperactivity in the BLA, concurrently inducing structural atrophy and functional suppression in the superficial layers of the PFC [132]. Hyperexcitability of the BLA, mediated via its projections to the PFC, may impair the top-down inhibitory regulation of the HPA axis by the PFC, ultimately resulting in disrupted HPA axis negative feedback and sustained elevation of glucocorticoid levels [132]. In a depression model induced by neonatal clomipramine administration, these pathological changes are manifested simultaneously as BLA volumetric enlargement, elevated plasma corticosterone levels, and associated anxiety-like behaviors [133]. Notably, these abnormalities can be reversed by stimulation targeting the reward circuitry [133]. Collectively, these findings underscore that correcting the hyperactive state of the BLA–PFC–HPA axis circuit represents a crucial pathway for restoring emotional homeostasis.

However, a comprehensive understanding of HPA axis regulation necessitates moving beyond the macroscopic neural circuit level to the cellular and microenvironmental homeostatic levels. The role of arginine vasopressin (AVP) and its potential regulation of aquaporins offer a novel perspective. AVP is a critical regulator of the HPA axis [134]. Its action via the V1b receptor in the anterior pituitary potently activates the HPA axis, a process highly correlated with specific depressive phenotypes, such as depression comorbid with anxiety and psychomotor retardation [135]. Beyond this classical endocrine pathway, the role of AVP in regulating water and ion homeostasis within the central nervous system is garnering increasing attention [136]. AVP is synthesized and released by the suprachiasmatic nucleus (SCN), the master circadian pacemaker [137,138]. AVP neurons within the SCN have been identified as key integrators of time- and osmoregulation-based cues [139]. Research indicates that SCN AVP neurons can be activated via a unique GABA-dependent excitatory mechanism in response to osmotic changes, triggering adaptive responses specific to circadian timing [140]. Given that HPA axis activity exhibits a robust circadian rhythm, and its dysregulation is a core feature of depression, the rhythmic output from the SCN is essential for modulating upstream HPA axis regions, including the paraventricular nucleus (PVN).

Herein, we posit a testable scientific hypothesis: AVP derived from the SCN may influence neuronal excitability and rhythmic synchronization within HPA axis-related brain regions (including the SCN itself and the PVN) by modulating local water and ion homeostasis, thereby participating in the overall regulation of HPA axis activity. In this hypothetical framework, aquaporin-4 (AQP4) may play a pivotal effector role, given its crucial physiological functions in the central nervous system. Theoretically, osmotic changes triggered by SCN neuronal activity or local AVP release could alter extracellular space volume and ion concentration by affecting AQP4-dependent transcellular water transport [141]. Such hydrodynamic alterations may further impair astrocytic glutamate reuptake efficiency, leading to an imbalance in glutamatergic tone within the PVN and other related regions [91]. This could result in aberrant activation of CRH/AVP neurons and subsequent HPA axis hyperactivity [142]. Therefore, AVP may directly regulate the excitability threshold of HPA axis-related neurons at the cellular microenvironment level by modulating AQP4 function, thereby enabling multi-level regulation of HPA axis activity in concert with macroscopic neural circuits such as the BLA–PFC pathway.

Although existing literature has not directly confirmed AVP-mediated HPA axis regulation via AQP4, this hypothesis integrates the well-established functions of AVP in osmoregulation and circadian rhythm with the fundamental role of AQP4 in maintaining neural microenvironmental homeostasis. It provides a novel and potential cellular mechanistic framework for understanding HPA axis dysfunction under chronic stress. Future research should test this hypothesis in animal models, investigating whether alterations in hypothalamic AVP signaling correlate with changes in AQP4 expression or function in depression models, and whether modulating AQP4 can regulate HPA axis activity and depression-like behaviors. This line of inquiry may reveal a novel pathway connecting neuroendocrine signaling, fluid homeostasis, and neuronal excitability, potentially offering new targets for mechanistic research and therapeutic development in depression.

## 6. AQP4 and Antidepressive Treatment

### 6.1. Drug Treatment

Major antidepressants, including tricyclic antidepressants, selective serotonin reuptake inhibitors (SSRIs), and serotonin-noradrenaline reuptake inhibitors, exert pharmacotherapeutic effects are often accompanied by changes in AQP4 expression or localization (Table 3). Chronic stress downregulates hippocampal and prefrontal AQP4 expression, a pathology reversed by fluoxetine and escitalopram via Src kinase-dependent phosphorylation of AQP4 at Ser111, enhancing its membrane trafficking and orthogonal array assembly [73,143,144]. Structurally, fluoxetine binding to the σ1 receptor-astrocyte microdomain triggers Ca^2+^-dependent calmodulin activation, promoting AQP4 oligomerization and perivascular endfoot anchoring—processes abolished in AQP4-KO models [145]. Critically, AQP4 is indispensable for SSRI efficacy: fluoxetine fails to rescue hippocampal neurogenesis or volumetric deficits in AQP4-KO mice, as evidenced by persistent dendritic spine loss and impaired brain-derived neurotrophic factor (BDNF)-mechanistic target of rapamycin (mTOR) signaling in dentate gyrus granule neurons [146]. Novel glutamatergic agents similarly show effects that correlate with the normalization of AQP4 dynamics. For example, ketamine rapidly normalizes CUMS-suppressed AQP4 through HIF-1α-mediated transcriptional upregulation, facilitating astrocyte process elongation prior to synaptic protein recovery [76,147]. In addition, memantine profoundly reduces AQP4 and astrocyte loss, and attenuated demyelination and axonal loss in the spinal cord of mice which had received AQP4-IgG [148].

**Table 3:** Antidepressants associated with AQP4/astrocytes.

Drug Name	Drug Class	Research Phase	Effect on AQP4/Astrocytes	References
Ketamine	NMDAR antagonist	Marketed	Increased the number of GFAP-positive cells and upregulated AQP4 expression in the hippocampus of CUMS mice.	[76]
Ketamine	NMDAR antagonist	Marketed	AQP4-KO enhances hypnotic sensitivity to ketamine, shortens latency and prolongs duration of loss of righting reflex.	[149]
S-ketamine	NMDAR antagonist	Marketed	Significantly mitigated reactive astrocytosis in the hippocampal CA1 region, as indicated by reduced GFAP intensity and a decreased total number of intersections in Sholl analysis. Reduced the proportion of neurotoxic A1-type astrocytes (GFAP/C3-positive) and increased the proportion of neuroprotective A2-type astrocytes (GFAP/S100A10-positive).	[150]
Memantine	NMDAR antagonist	Marketed	Attenuates AQP4-IgG-induced loss of AQP4 and GFAP (astrocyte marker) in spinal cord white matter; Reduces astrocytic apoptosis.	[148]
Sertraline	SSRI	Marketed	Induces intracellular Ca^2+^ overload, mitochondrial membrane hyperpolarization followed by collapse, ROS generation, and caspase-3/PARP activation, leading to apoptosis in astrocytes.	[151]
Paroxetine	SSRI	Marketed	Induces intracellular Ca^2+^ overload, mitochondrial membrane hyperpolarization followed by collapse, ROS generation, and caspase-3/PARP activation, leading to apoptosis in astrocytes.	[151]
Fluoxetine	SSRI	Marketed	Reversed CMS-induced upregulation of hippocampal AQP4 expression; Enhanced proliferation of AQP4-positive adult neural stem cells in vitro.	[143]
Mirtazapine	Noradrenergic and specific serotonergic antidepressant (NaSSA)	Marketed	Promotes astrocyte proliferation and upregulates the antioxidant protein metallothionein (MT-1/2) expression in striatal astrocytes via astrocytic 5-HT1A receptors.	[152]
Vortioxetine	Serotonin partial agonist reuptake inhibitor (SPARI)	Marketed	Subchronic administration suppresses basal and hemichannel-activated astroglial L-glutamate release.	[153]
Vortioxetine	Serotonin Modulators and Stimulants (SMS)	Marketed	Significantly increased the number of ALDH1L1-positive astrocytes in the CA2/3 and CA1 hippocampal subregions.	[154]
Lithium	Mood stabilizer	Marketed	Attenuates stress-induced reduction in hippocampal AQP4 density in astrocytes (AQP4/GFAP ratio), particularly in the dentate gyrus; prevents decrease in AQP4 immunoreactive astrocyte coverage under stress conditions.	[75]
Omega-3 PUFA	Neuroprotective agent	Clinical Research	Suppresses TBI-induced upregulation of AQP4 expression (both protein and mRNA) and partially prevents loss of AQP4 polarity in astrocytes; Preserves AQP4-dependent glymphatic clearance function.	[155]
Agomelatine	Melatonergic agonist (MT1/MT2 receptor agonist) and 5-HT_2_c receptor antagonist	Marketed	Promotes AQP4 polarization at astrocytic endfeet, enhancing perivascular localization; Increases expression of AQP4 at perivascular endfeet in MPTP-induced PD mice; Enhances glymphatic influx and efflux, indicating improved AQP4-dependent fluid clearance.	[156]

Some mood stabilizers, such as Lithium, PUFA, and valproic acid (VPA) also share a common downstream effect of influencing AQP4 function to alleviate depressive symptoms. Lithium reverses stress-induced AQP4 reduction in the dentate gyrus, thereby attenuating hippocampal blood–brain barrier/neurovascular unit disruption, which may in turn restore neurogenesis and alleviate anhedonia [75]. Complementarily, PUFA restores the underlying glymphatic system disruption and protecting cerebral vascular function by enhances AQP4 membrane trafficking in prefrontal astrocytes, which is involved in its antidepressive effects [73]. Notably, according to Davoudi S et al., VPA counteracts AQP4 depolarization through its suppression of MMP-9, which cleaves β-dystroglycan and causes the depolarization [66]. These findings establish AQP4 as a common downstream effector or modulator influenced by diverse antidepressant agents, rather than a direct molecular target of these drugs. However, while these studies imply that AQP4 could serve as a potential direct target for novel antidepressant therapies, significant challenges must be acknowledged. Direct pharmacological targeting of AQP4 would need to address issues such as achieving cellular specificity for astrocytes, mitigating the risk of disrupting water homeostasis and inducing cerebral edema, and accounting for the regional heterogeneity of AQP4 expression and function across different brain areas.

### 6.2. Non-Drug Treatment

As a non-drug way, physical exercise confers antidepressant efficacy which involves AQP4-dependent neuromodulation, with high-intensity interval training (HIIT) restoring perivascular AQP4 polarization to enhance glymphatic clearance—evidenced by increased CSF tracer influx and orthogonal array density in hippocampal astrocytes [157]. Treadmill preconditioning attenuates cerebral edema via transcriptional AQP4 downregulation, while HIIT accelerates amyloid-β clearance through meningeal-lymphatic coupling [157,158]. Critically, exercise upregulates BDNF-TrkB-AQP4 signaling cascades, triggering P2Y1R-mediated ERK1/2 phosphorylation that reorganizes orthogonal arrays within astrocytic endfeet-a structural prerequisite for glymphatic competency [159]. AQP4 deficiency ablates exercise benefits: AQP4-KO mice exhibit abolished cognitive improvement, impaired glymphatic flux, and blunted astrocytic lactate shuttle that deprives neurons of plasticity substrates [160]. These findings converge on AQP4 as an exercise-sensitive effector regulating protein homeostasis and metabolic resilience, with optimized exercise regimens demonstrating superior AQP4 activation versus moderate continuous training in treatment-resistant cohorts.

Beyond exercise, diverse non-pharmacological interventions ameliorate depressive pathology through mechanisms that ultimately involve AQP4-mediated neuromodulation. Electroacupuncture, an effective intervention for depression, has been shown to attenuate astrocyte damage by downregulating AQP4 expression, a process mediated through the reduction in METTL3-dependent m6A methylation on lncRNA MALAT1 [161]. Phototherapy enhances perivascular AQP4 polarization in the suprachiasmatic nucleus, synchronizing circadian glymphatic clearance of neuroinflammatory cytokines via melanopsin-astrocyte signaling [162]. Slow-wave sleep enhancement triples glymphatic influx by augmenting astrocytic Ca^2+^ waves and AQP4-dependent paravascular flow, correlating with reductions in hippocampal TNF-α and restored HPA axis rhythmicity [163]. These findings position AQP4 as a key downstream mediator or convergent node through which a spectrum of non-pharmacological mechanisms alleviate depressive pathology, highlighting its role in the final common pathway of these interventions rather than as a primary target. This further underscores that while AQP4 modulation is a crucial component of the therapeutic response, the development of strategies aimed directly at AQP4 would require careful consideration of its complex physiological roles, including the precise spatiotemporal control needed to avoid adverse effects such as edema and to respect the functional heterogeneity of AQP4 across neurocircuits implicated in depression.

## 7. Conclusions and Future Perspectives

**Figure 1:**
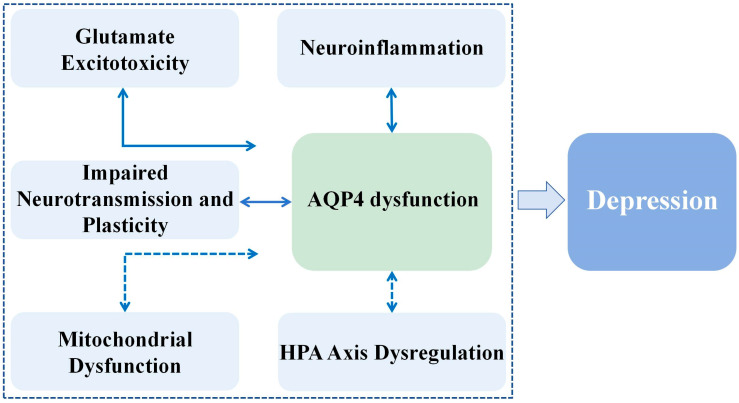
This figure illustrates how AQP4 dysfunction contributes to the pathophysiology of dysregulated astrocytic-neuron-immune network through multiple pathways, including the development of depression: (1) glutamate excitotoxicity: AQP4 dysfunction is directly associated with impaired glutamate clearance from astrocytes, resulting in elevated synaptic glutamate and neuronal toxicity in preclinical studies; (2) Neuroinflammation: AQP4 deficiency or mislocalization has been shown to exacerbate glial cell activation and proinflammatory cytokine release in animal models of depression; (3) impaired neurotransmission and plasticity: AQP4-mediated changes in extracellular homeostasis and neuroinflammation may secondary to impaired synaptic plasticity and monoaminergic transmission; (4) Mitochondrial dysfunction: The role of AQP4 in ion-water homeostasis may indirectly affect astrocyte bioenergetics and oxidative stress, and this pathway is under active investigation; (5) HPA axis dysfunction: AQP4 dysfunction induced by chronic stress may impair the clearance of neuroactive hormones and cytokines, which may lead to glucocorticoid resistance and persistent HPA axis hyperactivity. Arrow notations: Solid arrows indicate established evidence based on experimental data; dashed arrows represent hypothetical or proposed pathways requiring further validation.

This review establishes AQP4 as a pathophysiological node in depression, implicated in a spectrum of core pathological cascades: glutamate excitotoxicity, impaired neurotransmission, neuroinflammatory, mitochondrial dysfunction, and HPA axis dysregulation (Figure 1). Furthermore, AQP4 appears to mediate, at least in part, the therapeutic effects of certain pharmacological and non-pharmacological interventions, highlighting its functional relevance in treatment responses. While the majority of supporting data are currently associative or derived from global knockout and chronic stress models, the collective evidence positions AQP4 not as a sole causative factor but as a critical contributing and permissive element within the broader astrocyte–neuron–immune network. Its dysfunction likely facilitates disease progression by disrupting water homeostasis, neurovascular coupling, and glymphatic clearance, thereby creating a permissive environment for the convergence of multiple depressive pathomechanisms.

Despite extensive research efforts carried out to date, the mechanisms linking AQP4 to depression remain largely unclear. Four transformative directions will advance AQP4-targeted precision psychiatry: ① Nanoscale Dynamics: Resolve AQP4 OAP reorganization in live astrocytes using cryo-electron tomography to define drug-binding pockets for orthogonal array stabilizers [164]. ② Circadian Biology: Track diurnal AQP4 redistribution via intravital two-photon imaging [165] to determine its role in diurnal mood variation. ③ Circuit-Specific Delivery: Develop astrocyte-targeted adeno-associated virus vectors for region-restricted AQP4 overexpression in treatment-resistant models [166]. ④ Multi-Omic Integration: Decode comorbid mechanisms through spatial transcriptomics of perivascular niches in depression with neurodegeneration/vascular disease [125,167].

## Data Availability

No new data were created or analyzed in this study. Data sharing is not applicable to this article.

## Abbreviations

The following abbreviations are used in this manuscript:

AQP4	Aquaporin-4
CNS	Central nervous system
MDD	Major depressive disorder
AD	Alzheimer’s disease
OAPs	Orthogonal arrays of particles
CSF	Cerebrospinal fluid
CUMS	Chronic unpredictable mild stress
AQP4-KO	AQP4 knockout
LPS	Lipopolysaccharide
AAV5	Adeno-associated virus 5
CUS	Chronic unpredictable stress
AQP4-ON	Aquaporin-4 antibody-seropositive optic neuritis
ION	Idiopathic ON
CAD	Coronary artery disease
PUFA	Polyunsaturated fatty acids
MDE	Major depressive episode
BD	Bipolar disorder
Gd-DTPA	Gadolinium-diethylenetriamine pentaacetate
SED	Stress-induced exhaustion disorder
HCs	Healthy controls
GFAP	Glial fibrillary acidic protein
CMS	Chronic mild stress
ELS	Early-life stress
MS	Maternal separation
HFD	High-fat diet
TBI	Traumatic brain injury
NMDAR	N-methyl-D-aspartate receptor
LTP	Long-term potentiation
NF-κB	Nuclear factor κB
IL-1β	Interleukin-1β
TNF-α	Tumor necrosis factor-α
HPA	Hypothalamic–pituitary–adrenal
BLA	Basolateral amygdala
AVP	Arginine vasopressin
SCN	Suprachiasmatic nucleus
PVN	Paraventricular nucleus
SSRIs	Selective serotonin reuptake inhibitors
BDNF	Brain-derived neurotrophic factor
mTOR	mechanistic target of rapamycin
VPA	Valproic acid
HIIT	High-intensity interval training
